# Combined with multimodal medical imaging and artificial intelligence for early diagnosis of Parkinson's disease

**DOI:** 10.3389/fneur.2025.1650968

**Published:** 2025-10-01

**Authors:** Sitong Lu, Shuang Gui, Chunyun Wang, Yu Liu

**Affiliations:** ^1^Department of Radiology, Chongqing Public Health Medical Center, Chongqing, China; ^2^School of Medicine and Life Sciences, Chengdu University of Traditional Chinese Medicine, Chengdu, Sichuan, China; ^3^Department of Radiology, Qianjiang Central Hospital of Chongqing, Chongqing, China; ^4^Medical Imaging Department, Beijing Anzhen Nanchong Hospital of Capital Medical University & Nanchong Central Hospital, Nanchong, Sichuan, China

**Keywords:** Parkinson's disease, early diagnosis, multimodal medical imaging, artificial intelligence, MRI

## 1 Introduction

Parkinson's disease (PD), also known as “shaking palsy,” is the second most common neurodegenerative disease in the world. Its main symptoms are divided into motor: static tremor, bradykinesia, postural balance disorder, and myotonia (1); and non-exercise: constipation, memory loss, frequent urination, urgent urination, depression, decreased sense of smell (2), etc. Epidemiological data show that both PD incidence rate and prevalence rate show a significant age-dependent growth trend (3, 21, 25). Male incidence rate is 1.5 times higher than female (29). Although the average survival time of patients after diagnosis is long and most of them die due to complications, due to the significant difficulties in early diagnosis, most patients have irreversible loss of dopaminergic neurons in substantia nigra when they are diagnosed. Limited by the existing medical cognition and technical level, there is no radical cure for Parkinson's disease at present. Clinical treatment still takes symptomatic intervention and delaying disease progress as its core goal. With the acceleration of global human-aging process, the number of Parkinson's disease patients continues to rise, which brings severe challenges to medical resource allocation and long-term care system. Therefore, achieving early and precise diagnosis of Parkinson's disease is of great clinical significance for improving the cure rate of the disease, prolonging the survival period of patients and enhancing their quality of life.

The current clinical diagnosis of Parkinson's disease mainly relies on typical symptom recognition, but this diagnosis method based on subjective symptom description of patients has significant limitations. The heterogeneity of symptoms between individuals can easily lead to missed diagnosis and misdiagnosis. In addition, the early symptoms of Parkinson's disease are hidden and some symptoms overlap with nervous system diseases such as multi-system atrophy and progressive supranuclear palsy (PSP), which further aggravates the difficulty of diagnosis. Although multimodal medical imaging techniques such as ultrasound computed tomography (CT), magnetic resonance imaging (MRI) have been applied to the diagnosis of Parkinson's disease, due to the lack of specific biology markers, the diagnosis based solely on conventional imaging features is still highly dependent on clinical experience. This diagnostic mode not only has insufficient sensitivity and poor stability, but also often has atypical imaging manifestations, which is difficult to meet the precise needs of early diagnosis. Finding an objective and accurate inspection method is a problem to be solved at present.

With the rapid development of artificial intelligence technology (22), the medical auxiliary diagnosis system model based on multimodal images has opened up a new path for the early diagnosis of Parkinson's disease. Deep mining of massive medical image data through advanced algorithms such as deep learning and convolutional neural network can effectively identify atypical microscopic image features of early Parkinson's disease and significantly improve diagnostic efficiency and accuracy (4, 30). Compared with traditional diagnosis methods, artificial intelligence-assisted diagnosis system shows higher stability and reliability, which provides an objective and quantitative basis for clinical decision-making.

In view of the key position of early diagnosis in the treatment of Parkinson's disease, the core of breaking through the bottleneck of existing diagnostic technology lies in integrating multimodal medical imaging technologies such as Positron emission tomography (PET), MRI, and single photon emission computed tomography (SPECT) (28, 32). Each imaging technology can obtain disease information from metabolism, structure, function and other dimensions based on different principles. Through technological integration, complementary advantages can be achieved, which is helpful for comprehensively capturing disease characteristics. On this basis, an early diagnosis auxiliary model framework based on artificial intelligence is constructed to deeply analyze the potential features in multimodal image data through optimization algorithms, which is expected to further improve the accuracy and stability of diagnosis. By combining multimodal medical imaging and artificial intelligence technology, it provides clinicians with efficient and reliable intelligent decision-making tools to promote the development of accurate and intelligent early diagnosis of Parkinson's disease.

## 2 Current status of multimodal medical imaging artificial intelligence-assisted early diagnosis of Parkinson's disease

In recent years, the cross-fusion of artificial intelligence and medical images has brought new breakthroughs for early diagnosis of PD. For example, rediomics can extract quantitative features such as texture and shape from medical images with high-throughput manner, which are invisible to the naked eye, and transform images into analyzable data matrices; artificial intelligence algorithms, especially deep learning models, can deeply mine these high-dimensional features to achieve accurate pattern recognition.

### 2.1 Research progress of artificial intelligence combined with MRI in PD diagnosis

As a high-resolution structural imaging technology, magnetic resonance imaging (MRI) can provide high-resolution three-dimensional images of patients' brains, which helps to enhance the interpretability of models (33). For example, the significance map of convolutional neural network (CNN) generated by Camacho et al. (5) through MRI images, with an area under the receiver operating characteristic curve (AUC-ROC) of 0.87, clearly reveals the key function of frontal cortex and several deep gray matter structures in the diagnosis of early Parkinson's disease. However, similar to other studies based on retrospective data, this study has the problem of heterogeneity of inclusion/exclusion criteria and overall diagnostic criteria for Parkinson's disease, which may weaken the adaptability between early characteristic clinical picture and diagnostic criteria for Parkinson's disease. With the continuous development of imaging omics technology, the clinical diagnosis process has gradually evolved to carry out targeted multimodal MRI evaluation after routine MRI examination. The fused multimodal MRI data can provide effective support for the specific diagnosis of early Parkinson's disease with the advantage of machine learning algorithm in big data integration analysis. Chougar et al. (6) used MRI data to build a machine learning algorithm, and successfully distinguished Parkinson's disease (PD), progressive supranuclear palsy (PSP) and multi-system rhomboid contraction (MSA), especially in distinguishing PD from MSA. However, MRI misdiagnosis was also found during the study. Therefore, in the follow-up study, high-field MRI or dynamic enhanced MRI technology can be explored to further tap the potential of multimodal MRI combined with artificial intelligence in the specific diagnosis of Parkinson's disease. In addition, Ye et al. (7) used two different structural MRI (sMRI) sequences (T2-FLAIR and T1WI) to build an imaging model, and achieved AUC of 0.896 and 0.899, respectively, which confirmed the good clinical practicality of the model through decision curve analysis. Pahuja and Prasad (8) focused on the optimization of model framework, and explored PD classification performance under different frameworks based on T1-weighted MRI and single photon emission computed tomography (SPECT) images. Based on the above studies, it is not difficult to find that the clinical effect of Chougar et al. (6) may not be satisfactory in the case of classification characteristics. However, when meeting the clinical effect, such as Ye et al. (7), the data limitations are relatively large; As for the study by Camacho et al. (5), although it also has clinical limitations, thanks to the introduction of CNN, it is possible to determine high-precision markers for specific regions and enhance the interpretability of the model. Therefore, MRI, as a high-resolution structural imaging technique, can enhance the interpretability of the model, but there is a problem of heterogeneity in diagnostic criteria in retrospective studies; meanwhile, MRI technology can assist in the classification of different diseases and subtypes, but it is also limited by clinical application. With the development of imaging omics, multimodal MRI evaluation has become a clinical trend, and its fusion data combined with machine learning algorithm can effectively assist early diagnosis of Parkinson's disease.

### 2.2 Research progress of artificial intelligence combined with PET/MRI in PD diagnosis

One of the core strategies for accurate diagnosis of early Parkinson's disease is to explore its characteristic differences from similar diseases. Magnetic resonance imaging (MRI) is often used to assist in the classification of Parkinson's disease subtypes due to its ability to image high-resolution anatomical structures. Positron emission tomography (PET) imaging technology can quantify the function of dopaminergic system and glucose metabolism pattern, and provide specific biology markers. The multimodal fusion of the two not only realizes the correlation analysis between function and structure, but also significantly improves the diagnostic accuracy, making PET-MRI technology an important tool to identify similar neurodegenerative diseases. Sun et al. (9) showed that the constructed multimodal model outperformed the single-modal model regardless of how PET and MRI data were combined and sequenced. The developed PET/MRI radiomics-clinical combined model achieved an area under the receiver operating characteristic curve (AUC) of 0.993, fully demonstrating the significant potential of this technology in the clinical differentiation of Parkinson's disease (PD) and multiple system atrophy (MSA). Another study focused on [18F] FDG PET/MRI (10), by training an artificial intelligence model integrating metabolic, structural and functional information, confirmed that the comprehensive imaging model was significantly superior to the simple clinical diagnosis model in distinguishing PD from MSA, which mutually confirmed the conclusions of Sun et al. (9). However, the study also points out that although automatic region of interest segmentation improves efficiency, its accuracy is still not as good as manual labeling, and finding a more accurate automatic sketching algorithm is the key to achieve fully automated diagnosis. Silva-Rodríguez et al. (11) used [18F] FDGPET/MRI technology to evaluate the effectiveness of structural MRI (sMRI) and diffusion magnetic resonance imaging (dMRI) assisted by machine learning algorithms in detecting mild cognitive impairment (PD-MCI) and dementia (PDD) in Parkinson's disease, and found that dMRI has more advantages in revealing microstructural changes in early brain regions of patients. It is worth noting that neither of the first two studies included the gold standard of pathological results. Sun et al. (9) conducted a retrospective study, sample heterogeneity cannot be ruled out. Hu et al. (10) also cannot guarantee the accuracy of automatic segmentation. While the third study (11) lacked control group and follow-up data, and only conducted a cross-sectional study. To sum up, in order to fully utilize the application value of the hybrid PET/MRI technology in the early diagnosis of Parkinson's disease, it is urgent to establish a large-scale and long-term follow-up control database, providing solid data support for the feature extraction and optimization of artificial intelligence models.

### 2.3 Research progress of artificial intelligence combined with PET/CT to assist PD diagnosis

At present, it is believed that the main cause of Parkinson's disease is the loss of dopaminergic neurons in substantia nigra. For early Parkinson's patients (23), the new PET/CT technology has higher resolution on minor changes in small lesions (24), such as dopamine transporter (DAT) or presynaptic membrane vesicle monoamine transporter (31, 34), and is more sensitive to striatal dopaminergic neurons. Combined with artificial intelligence technology, it can help ultra-early Parkinson's diagnosis. Comte et al. (12) used [18F] FDOPA PET/CT to mark scans with or without dopaminergic neurons, and selected biology markers to build a regression model. In the external test set, the study achieved an AUROC of 0.96, confirming the potential of PET/CT imaging techniques in combination with artificial intelligence to identify early Parkinson's dopaminergic neurons. However, this research has not undergone clinical trials, and the specific performance of the model is also uncertain due to the influence of the research methods. Seo et al. (13) analyzed the changes of striatal dopamine transporter (DAT) uptake in PD by PET/CT imaging technology, and found that DAT uptake was related to the decrease of glucose metabolism in brain region, while only some visual functions were significantly affected by DAT, and DAT uptake decreased in the order of PP AP and caudate nucleus, and then the average standard uptake ratio decreased, resulting in visuospatial cognitive dysfunction. However, this study is still retrospective, and the control of some variables and the universality of the results are not up to the expected level. We also learned that, as mentioned in the Wu et al. (14) study, the 3D parameters of the new striatum of PD patients included in the study based on good consistency of quantitative parameters between 11C-CFT PET/CT planar and 3D images are more associated with disease progression than planar parameters, providing another potential evidence for future Parkinson's diagnosis.

### 2.4 Summary

With the rapid iteration of artificial intelligence technology, intelligent diagnostic models based on multimodal medical images have become an important research direction for early identification of Parkinson's disease (PD), and new high-performance models are constantly emerging. However, the current single medical imaging technology still has significant limitations in clinical application: although computed tomography (CT) and magnetic resonance imaging (MRI) are widely used, they lack specificity in detecting the loss and dysfunction of tiny neurons in the early stage of PD, and their imaging markers tend to overlap with other neurodegenerative diseases. Although functional magnetic resonance imaging is highly sensitive to changes in brain function, it is difficult to achieve large-scale clinical promotion because of its complex technical operation and strong device dependence. Positron emission tomography (PET) and single photon emission computed tomography (SPECT) can visually reflect the functional status of dopaminergic system in the brain, but their clinical application is limited due to high examination cost, radiation exposure risk and low popularity of equipment. In view of the above technical bottlenecks, building an artificial intelligence model integrating multimodal image data has become a breakthrough direction. Based on the existing multimodal combinations such as MRI-PET, MRI-SPECT and PET-CT, it is expected to achieve significant improvement in model stability and diagnostic accuracy by deeply integrating complementary information of different imaging technologies (such as high-resolution anatomical structure of MRI and metabolic and functional specificity of PET/SPECT) (20). This unified multimodal model can not only enhance the recognition ability of early pathological features of PD, but also provide richer and more representative data sets for model verification, thus providing reliable technical support for early accurate diagnosis of PD.

## 3 Our thoughts

Based on the development status and bottleneck of multimodal artificial intelligence model in the field of early diagnosis of Parkinson's disease, building an end-to-end intelligent screening system for early Parkinson's disease has become an important path to realize visual intelligent diagnosis and decision-making in the whole clinical process. At the same time, relying on large-scale Parkinson's patient queue data for deep learning model training and digging up potential disease characteristic patterns, it is expected to find biology markers with high specificity and sensitivity for early diagnosis. By optimizing the algorithm architecture and model parameters, we can promote the transformation of Parkinson's disease diagnosis and treatment mode to intelligence and precision, and bring new hope for overcoming early diagnosis problems and improving patient prognosis.

### 3.1 Organically integrate multimodal images

In the early diagnosis of Parkinson's disease, the multimodal imaging fusion task such as MRI, PET, CT can be systematically divided into four core steps. The first is the acquisition and preprocessing of raw data, converting the collected data into the Digital Imaging and Communications in Medicine (DICOM) standard format, verifying the integrity of metadata, and performing denoising processing for PET and CT data; using rotation translation, ANTs, etc. to achieve spatial registration, bridging resolution differences, and enhancing image contrast to highlight edge features. The second is the feature fusion stage, adopting multi-dimensional fusion strategies: directly superimposing MRI and PET images through image registration; combining MRI volume features with PET metabolic features and other different modal features; using deep learning algorithms to achieve more complex feature fusion.

Based on the above content, further supplements are given. It is recommended that the selection parameters of the spatial registration threshold be set to rigid registration: the maximum mutual information error threshold was 0.3 bits to balance accuracy and efficiency and nonlinear registration were adopted, and the original RMSE root mean square error threshold was < 2.0 mm (15) to meet the resolution requirements of substantia nigra nucleus in Parkinson's diagnosis. In addition, it is recommended to use third-order B-spline interpolation to preserve image texture to help better identify specific markers. Based on existing literature (16, 17) and theoretical analysis in this paper, we recommend using a deep learning-based U-Net architecture to process PET images, with input as 4D dynamic time frames, and the loss function employing Poisson and SSIM weighted (weight ratio of 0.7:0.3) to better preserve functional metabolic texture. Additionally, a multi-channel local attention module is developed for feature extraction from CT images. A five-fold stratified cross-validation strategy is adopted, along with the NAdamW optimizer configuration, to avoid training instability caused by differences in gradient magnitudes between modalities. Since clinical trials have not been carried out in this paper, the real parameters are expected to be supplemented and improved in real applications.

### 3.2 Establish an end-to-end artificial intelligence model for early diagnosis of Parkinson's disease based on multimodal images

The construction of an end-to-end intelligent early Parkinson's diagnosis model based on standardized multimodal image data can be promoted according to the following processes. First, feature extraction of multimodal data is carried out, and a combination of manual and deep learning is adopted. The CT ventricular volume and PET standard uptake values were extracted manually, and the global features of each mode were obtained by using the pre-training model. With the help of Python library, 3D Slicer, MONAI and other tools, various types of radiological features such as shape, texture and functional metabolism can be extracted. Secondly, the model architecture is designed, and 3D CNN is selected to process volume image based on its ability to stably maintain good accuracy (18), Transformer to process multimodal sequences and global features, and a multi-branch fusion network is constructed. By designing key modules, multi-level image fusion is realized to form an end-to-end network in which the original data can be input and the diagnostic results can be output. Then, the model training and optimization are carried out, and the continuous and discrete features are randomly enhanced. The loss function and optimizer are selected, and the model with the best performance in the verification set is retained under the scenario of multi-center data sharing. Finally, the model is verified and iterated, taking AUC, sensitivity and accuracy as evaluation indicators, visualizing key areas to improve interpretability, and regularly fine-tuning according to new case data to realize dynamic update of the model and ensure continuous optimization of diagnostic efficiency.

### 3.3 Applying end-to-end models to clinical diagnosis of Parkinson's disease

The application of end-to-end intelligent diagnosis model in clinical auxiliary diagnosis of Parkinson's disease needs to be promoted from three aspects: visual integration, system docking and clinical verification. At the level of visual integration, by generating heat maps of MRI images, the key lesion areas such as iron deposition in the substantia nigra and density changes can be visually highlighted. After dimensionality reduction of multimodal image features, the distribution differences between healthy people and patients were displayed by scatter diagram, and the decision-making basis of the model was clearly presented. Moreover, by calculating the contribution values of each pattern to the final diagnosis and visual attention weights, the model's interpretability is enhanced to ensure the reliability of the diagnostic logic. For example, using GradientSHAP technology to generate heatmaps primarily focused on striatal dopamine transporter activity improves interpretability. The model shall be deeply integrated with the picture archiving and communication system (PACS) system during system docking. On the one hand, it ensures that the model can directly analyze standard DICOM format data and unify metadata cleaning rules of different manufacturers. On the other hand, encrypted communication and patient anonymity are adopted, only necessary metadata such as age and gender are reserved, data access rights are strictly restricted to authorized medical care and development teams, and operation logs are recorded. In addition, a doctor feedback channel is established to collect cases of misdiagnosis and missed diagnosis in real time, and the model is continuously optimized through automatic sample recovery function and regular performance report of PACS system. In the clinical verification stage, case samples can be collected from several large hospitals to verify the sensitivity and accuracy of the model through independent input data; or carry out controlled trials with traditional diagnostic methods, and use statistical means to evaluate the clinical value of the model, so as to provide a solid basis for its clinical promotion.

In fact, the aforementioned viewpoints still have deficiencies in clinical application, so we further improved the relevant mechanisms. First, retrospective data validation was conducted to check if the technical test imaging data can be seamlessly transmitted and to confirm its bidirectional synchronization with the RIS system. Second, prospective comparative studies were carried out, such as rolling deployment in three large hospitals each month, using real-time tracking of diagnostic decision-making paths through doctor group interviews. Finally, federated learning frameworks were utilized to achieve cross-institutional model optimization. Despite this, we still acknowledge the potential shortcomings of this deployment strategy.

We believe that the future early Parkinson's clinical diagnosis model will realize the automation and intelligence of the whole process to build a closed-loop system from data input to decision output. This will help clinicians to efficiently use artificial intelligence models to accurately judge the condition in PACS environment, and promote the diagnosis and treatment of Parkinson's disease to a new height.

## 4 Challenges of early diagnosis of Parkinson's disease based on artificial intelligence

At present, artificial intelligence models have shown great advantages in screening, diagnosis and treatment of early Parkinson's disease, but (26, 27) and challenges.

### 4.1 Interpretability of artificial intelligence models

Although artificial intelligence technology is widely used in the medical field at present, in order to convincingly prove the great potential of artificial intelligence in early Parkinson's diagnosis, a clearer and more visual data output interpretation system is needed, which is one of the difficulties faced by artificial intelligence at present. We can only effectively observe the input and final output results of data, how to get this result, and the unclear principle behind the result is like a “black box” (4), which also leads to people's difficulty in understanding the processing flow to a certain extent. Coupled with the small impact of changing the internal structure of the model on the algorithm performance, whether the artificial intelligence model can be widely used in the medical field depends on whether it can overcome the uninterpretability of the large algorithm model.

### 4.2 Standardized data collection and processing

In the early diagnosis of Parkinson's disease, standardized data acquisition and processing is the key foundation for building an efficient multi-modal artificial intelligence model. Different medical imaging technologies have significant differences in data acquisition stage. The imaging principles, equipment parameters and scanning protocols of CT, MRI, PET, and SPECT are different. In addition, the operation specifications and equipment models of different medical institutions are different, resulting in the lack of uniform standards for original data in terms of format, resolution and gray value, which brings great challenges to subsequent data integration and model training. In the process, data standardization process is particularly important. First of all, the original data must be converted into DICOM standard format to ensure the standardization of data storage and transmission. At the same time, strict quality control shall be carried out to eliminate unqualified data with excessive noise and obvious artifacts. Before multimodal data fusion, spatial registration techniques, such as affine transformation and nonlinear registration based on ANTs, must be used to eliminate the spatial positional deviations between different modal images, and interpolation algorithms should be adopted to unify the resolution. In addition, the quantitative data of radioactive tracers for PET and SPECT shall be standardized to eliminate measurement errors caused by different equipment and batches of tracers. Only by establishing a perfect standardized data acquisition and processing system can the stability and diagnostic efficiency of multimodal models be effectively improved.

### 4.3 Ethical and legal issues

As an emerging technology, artificial intelligence faces many ethical and legal challenges in the development process. Model training relies on the continuous updating of a large amount of data, but if the principle of informed consent is not strictly followed in data collection, it will easily lead to the risk of data abuse and leakage (19). At the same time, the lack of responsibility definition mechanism has become a prominent problem. When artificial intelligence technology generates benefits or causes damage, there is no clear legal basis for the division of responsibilities among developers, users and the system itself. In addition, at present, the regulation of artificial intelligence technology by international laws and regulations lags behind, and a perfect data tracing and supervision system has not yet been established. Under the traditional legal framework, the restraint mechanism for non-human subjects is almost blank, which leads to a large number of regulatory blind spots in artificial intelligence applications. These problems make the large-scale promotion of artificial intelligence in the medical and clinical field face great obstacles, and it is necessary to build a safe and compliant technology application environment through legal system innovation and ethical norms improvement.

### 4.4 Clinical realism

Unfortunately, the model proposed in this study is a conceptual framework and not yet be carried out in clinical trials, so the specific clinical application of the model is not clear. Future work will be dedicated to filling the gap between theoretical development and clinical application.

## 5 Conclusion

The continuous iteration of artificial intelligence technology has opened up a new path for early diagnosis of Parkinson's disease and brought hope for optimizing treatment schemes. Future research can deeply integrate artificial intelligence and multimodal medical imaging technology to build an end-to-end automatic early screening system. The system will realize seamless connection with the medical system and achieve intelligent visualization of the whole process from data collection to diagnosis decision. Through this efficient and convenient diagnosis mode, it is expected to promote the early diagnosis and treatment of Parkinson's disease, reduce the proportion of patients in the middle and advanced stage, and provide a new direction for overcoming the “incurable” problem of Parkinson's disease.
